# Rapid functionalisation of cell membrane‐coated nanoparticles using a modular approach

**DOI:** 10.1002/ctm2.1721

**Published:** 2024-06-20

**Authors:** Nishta Krishnan, Ronnie H. Fang, Liangfang Zhang

**Affiliations:** ^1^ Department of NanoEngineering Chemical Engineering Program, and Moores Cancer Center University of California San Diego La Jolla California USA; ^2^ Division of Host‐Microbe Systems and Therapeutics Department of Pediatrics University of California San Diego La Jolla California USA

**Keywords:** cell membrane coating, cellular nanoparticle, modular functionalisation, nanomedicine

1

Cell membrane‐coated nanoparticles (CNPs) have emerged as a powerful tool that can be used to address a range of biomedical challenges. CNPs leverage natural cell membranes as a cloaking layer for disguising synthetic nanoparticle cores, creating a biomimetic platform with enhanced biocompatibility and immune evasion characteristics.^1^ The membranes from various cell types have been used to create novel CNP formulations, with each displaying unique properties that enable it to excel at drug delivery applications.^2^ For example, by leveraging the inherent long circulation and immune‐evading properties of red blood cells, CNPs that improve the blood residence of systemically administered drug payloads have been developed.^3^ Platelets interact with different disease substrates and have been used to generate CNPs for treating cardiovascular diseases and bacterial infections.^4^ Tumour‐targeting properties can be bestowed to nanoparticles using membrane coatings derived from cancer cells, many of which naturally exhibit homotypic binding properties.^5^ The flexibility of the membrane coating approach has enabled CNPs to be generated using a variety of other cell types, including immune cells, stem cells, epithelial cells and bacteria.^6^ With the countless combinations of synthetic nanomaterials and membrane coatings that exist, highly functional nanoformulations with favourable properties for in vivo delivery can be readily developed for a wide range of applications.

By utilising approaches such as genetic manipulation, metabolic engineering, lipid insertion and membrane hybridisation, researchers have created modified cell membranes that further enhance the functionality of CNPs.^7^ In general, these modification strategies can be used to enrich and broaden nanoparticle function while preserving the natural benefits of the cell membranes. Notably, genetic engineering enables specific proteins or receptors to be upregulated or downregulated on source cells, and these edits are passed down onto the final membrane‐coated nanoparticles after CNP fabrication. Recently in *Nature Nanotechnology*, Krishnan and colleagues further expanded upon this concept to create a modular approach for biomimetic nanoparticle functionalisation (Figure 1).^8^ By genetically modifying the cell membrane component to express an adapter protein, it was demonstrated that the resulting CNPs could be rapidly equipped with different functional ligands.

**FIGURE 1:**
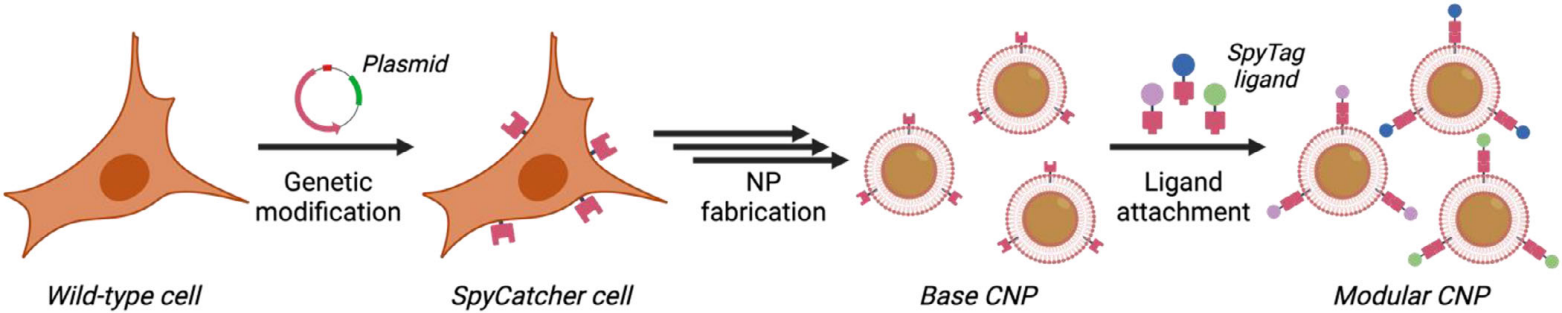
Cells are genetically engineered to express SpyCatcher on their surface. The membrane is then derived from the cells and coated onto synthetic nanoparticle cores. These CNPs can be modularly modified with SpyTag‐labelled ligands, resulting in novel CNPs that demonstrate enhanced functionality. Created with BioRender.

In order to generate the modular CNPs, source cells were first genetically altered to express a membrane‐anchored SpyCatcher protein, which can spontaneously form a covalent attachment with any ligand labelled with a short SpyTag peptide sequence.^9^ This engineered membrane was coated over a nanoparticle core fabricated using the biodegradable poly(lactic‐*co*‐glycolic acid). Once this base modular CNP formulation was generated, it could then be functionalised with various SpyTag‐labelled ligands​​. As an initial test, the nanoparticles were incubated with a SpyTag‐mKate2 fluorescent protein construct. The mKate2‐functionalised CNPs were similar in size and zeta potential compared with the base formulation, and they also exhibited a core–shell structure that is characteristic of CNPs. Subsequently, three model targeting ligands were selected, including a designed ankyrin repeat protein (DARPin) against mCherry, an affibody against epidermal growth factor receptor (EGFR), and a single‐chain variable fragment (scFv) against human epidermal growth factor receptor 2 (HER2). It was confirmed that the modular CNPs demonstrated affinity to cells with high surface expression of mCherry, EGFR or HER2 when functionalised with the corresponding ligand.

Finally, the modular CNPs were evaluated for their ability to specifically deliver payloads in vivo using a murine xenograft tumour model developed by subcutaneously implanting SKOV3 human ovarian cancer cells (EGFR^+^ and HER2^+^). Neither the base CNPs nor the DARPin‐functionalised CNPs targeting mCherry displayed any appreciable tumour targeting. In contrast, the affibody‐ and scFv‐functionalised CNPs targeting the two cancer markers overexpressed on the SKOV3 cells were able to efficiently localise to the tumour site. When used to deliver docetaxel as a model chemotherapeutic drug, these two formulations significantly slowed the rate of tumour growth and were able to more than double the median post‐treatment survival times. Importantly, all CNP formulations showed low systemic toxicity, and minimal adverse effects were observed.

The modular functionalisation approach presented in this work enables different ligands to be rapidly attached onto the surface of CNPs without the need to reengineer the source cells with every new formulation. Cell engineering is generally a cumbersome process; the use of a SpyCatcher adapter system avoids the intricacies related to protein folding, glycosylation and shuttling, as well as how protein expression can impact cell growth and/or viability. This significantly cuts down on development time and can enable a large number of ligands to be quickly and reliably screened. Another advantage is that both the SpyCatcher‐expressing CNPs and SpyTag‐labelled ligands can be fine‐tuned and optimised separately, thereby enhancing the robustness and reliability of each component prior to their combination. New CNP formulations can also be created on‐demand, which may be particularly useful in scenarios in which dynamic changes are required. It should be noted that this modular approach is also compatible with non‐protein ligands, as the SpyTag peptide can be conjugated onto small molecules, nucleic acids, and many others.

The translation of modular nanoparticles into the clinic will require several critical considerations to be met. First, it will be necessary to minimise the batch‐to‐batch variability of the base CNPs. Along these lines, master cell banks will need to be established, and the impact of continued culture over time will need to be assessed using detailed genomic, transcriptomic and proteomic analytical techniques. Regarding CNP synthesis, microfluidic systems could be employed to precisely control the conditions under which nanoparticles are formed, leading to improved uniformity. For the SpyTag‐labelled ligands, optimal conditions for their production, purification, and storage will need to be established. Finally, processes will need to be established to confirm successful CNP functionalisation and to remove unreacted components. In the future, it is envisioned that large libraries of CNPs and SpyTag‐labelled ligands can be generated. Novel CNP formulations with unique and enhanced functionalities could then be developed to help manage a diverse range of disease conditions.

## CONFLICT OF INTEREST STATEMENT

L.Z. holds equity interests in Cellics Therapeutics and Cello Therapeutics. The other authors declare no competing interests.
